# ATL-like overgrowth and clonal expansion of HTLV-1 infected CD25+ CD4+ T- lymphocyte in humanized-NOG mouse model

**DOI:** 10.1186/1742-4690-8-S1-A13

**Published:** 2011-06-06

**Authors:** Jun-ichi Fujisawa, Kenta Tezuka, Runze Xun, Mami Tei, Norihiro Takenouchi, Masakazu Tanaka

**Affiliations:** 1 Dept. Microbiology, Kansai Medical University, Moriguchi, Osaka, 570-8506, Japan

## 

Humanized mice (huNOG) established by the intra-bone marrow transplantation of NOG-SCID mouse with CD133+ hematopoietic stem cells purified from human cord blood were infected with HTLV-1 in vivo by peritonial injection of γ-ray-irradiated MT-2 cells in 3 to 4 months after transplantation.

While normal differentiation of human T lymphocytes was observed in the spleen of uninfected huNOG mouse, HTLV-1 infection increased the number of CD25+ CD4+ T- lymphocytes and resulted in the splenomegaly within several months. In the late period of infection, where almost all of the blood cells in the mouse were composed of infected human T-lymphocytes, cells with highly lobulated or flower-shaped nuclei appeared in the peripheral blood.

Inverse PCR analysis of provirus integration sites revealed the polyclonal infection in the early phase and the oligoclonal expansion of infected T cells mostly in the population of CD25+ CD4+ T- cells in the late phase. Since substantial amount of anti-Gag antibodies and Tax-specific CTLs were detected in the serum and the spleen of infected mice, respectively, the involvement of immune system against HTLV-1 was suggested in the clonal selection of HTLV-1 infected T-cells in this system.

Thus, the HTLV-1 infected huNOG mouse model should provide a valuable system for the analysis of ATL pathogenesis and the development of treatments against various HTLV-1 associated diseases. Results from the analysis of gene expression in HTLV-1 infected T-cells during the course of infection and the effects of in vivo administration of various anti-tumor or anti-viral drugs on the overgrowth of infected T-cells will be discussed.

